# Case Report: Potential role of selective venous sampling for liquid biopsy in complex clinical settings: Three case presentations

**DOI:** 10.3389/fgene.2023.1065537

**Published:** 2023-03-28

**Authors:** Vladimira Tichà, Gianluigi Patelli, Gianpaolo Basso, Aurelio Prino, Elena Repetti, Maria Grugni, Bruno Damascelli

**Affiliations:** ^1^ Department of Interventional Oncology, EMO GVM Centrocuore Columbus, Milan, Italy; ^2^ Department of Radiology, ASST Bergamo Est-Bolognini Hospital, Seriate, Italy; ^3^ School of Medicine and Surgery, San Gerardo Hospital, University of Milano-Bicocca, Monza, Italy; ^4^ Palliative Care Department and Hospice, University Hospital Maggiore Della Carità, Novara, Italy; ^5^ TOMA Advanced Biomedical Assays S.p.A, Busto Arsizio, Varese, Italy

**Keywords:** Liquid biopsy, ctDNA, venous sampling, pancreatic cancer, prostate cancer, glioblastoma

## Abstract

Tumor mutation profiling from a blood sample, known as liquid biopsy, is a reality that has already been approved for some cancers. This molecular diagnostic method complements tissue biopsy but is less invasive and therefore more easily applied, especially during tumor evolution. Its use should allow detection of residual disease, evaluation of treatment response or resistance, and selection of targeted treatments. However, implementation of liquid biopsy in routine clinical practice is hindered by unsolved issues, one of which is the scarcity of circulating tumor DNA in blood samples drawn from peripheral veins. To address this problem, we propose minimally invasive selective venous sampling from the region of interest, as used for some hormonal studies and for mapping of endocrine tumors. Intuitively, selective sampling should improve the sensitivity of liquid biopsy by avoiding the dilution of tumor biomarkers that occurs in the peripheral circulation. We report three cases that illustrate the potential utility of selective liquid biopsy in complex clinical settings, providing implications for diagnosis and treatment as well as for monitoring over time, disease localization, identification of drug resistance, and differential diagnosis.

## Introduction

Among the many biological markers shed into the circulation by solid and blood cancers, the most widely used for molecular analysis is circulating tumor DNA (ctDNA) (Aravanis et al., 2017). Multigene panel tests, some validated, allow various types of tumors to be identified through the mutations released during cell apoptosis and necrosis and other release mechanisms, including active secretion (Diaz and Bardelli, 2014; Meddeb et al., 2019; Sànchez-Herrero et al., 2022). At present, application of liquid biopsy is complex and is affected by tumor and host characteristics, sample collection procedures, DNA extraction methods, genetic sequencing and, finally, interpretation of results. The amount of ctDNA is minimal, especially for some cancers (Gatto et al., 2021), and high costs and long processing times are obstacles to routine use of liquid biopsy. We have developed a sampling procedure using percutaneous venous catheterization to draw blood directly from the specific region of interest. This technique avoids the dilution of biological markers that occurs in samples drawn from peripheral veins, thus increasing the yield of ctDNA. Selective venous sampling is a well-known technique still used for localizing endocrine disorders (Zhao et al., 2020).

## Case presentations

All three patients presented here underwent selective liquid biopsy (SLB), an outpatient procedure involving percutaneous catheterization, preferably *via* the femoral vein, under local anesthesia. Access is achieved freehand or under ultrasound guidance using an MPIS-505-SST micropuncture set (Cook Medical Inc., Bloomington IN) and placing a 4F introducer. Depending on the sampling site, a Merit 4F CB1 (Merit Medical Systems Inc, Utah, United States) or a Cordis Multipurpose 4 F MPA2 (Cordis Corporation Miami Lakes, FL. United States) catheter, each having a lateral hole near the tip, is placed over a hydrophilic guidewire. If necessary, the final position of the catheter is confirmed by injecting a small amount of contrast medium. As the catheter is withdrawn from the access point, a blood sample is obtained from the femoral vein. This serves as the peripheral sample, which is collected and processed in exactly the same way as the selective venous samples. The blood samples (8 mL) are drawn directly into STRECK Cell-Free DNA BCT^®^ collection tubes (Streck, Omaha, Nebraska, United States), which stabilize the cell-free DNA (cfDNA). Extraction of the circulating cfDNA is automated using the QIAsymphony Circulating DNA kit (QIAGEN, Hilden, D-40724 Germany). The cut-off in our laboratory is around 0.1 ng/μL. Genetic sequencing is done with the Illumina NextSeq 550 Kit QIAseq Tumor Mutational Burden Panels (Illumina, San Diego, CA 92122, United States) for Next-Generation Sequencing (NGS). The panel is capable of interrogating 486 genes with known associations with cancer in a single analysis. Bioinformatic analysis is done using CLC Genomics Workbench and Clinical CLC Genomics Workbench e Clinical Insight software (QIAGEN, Hilden, D-40724 Germany).

## Case 1

**FIGURE 1:**
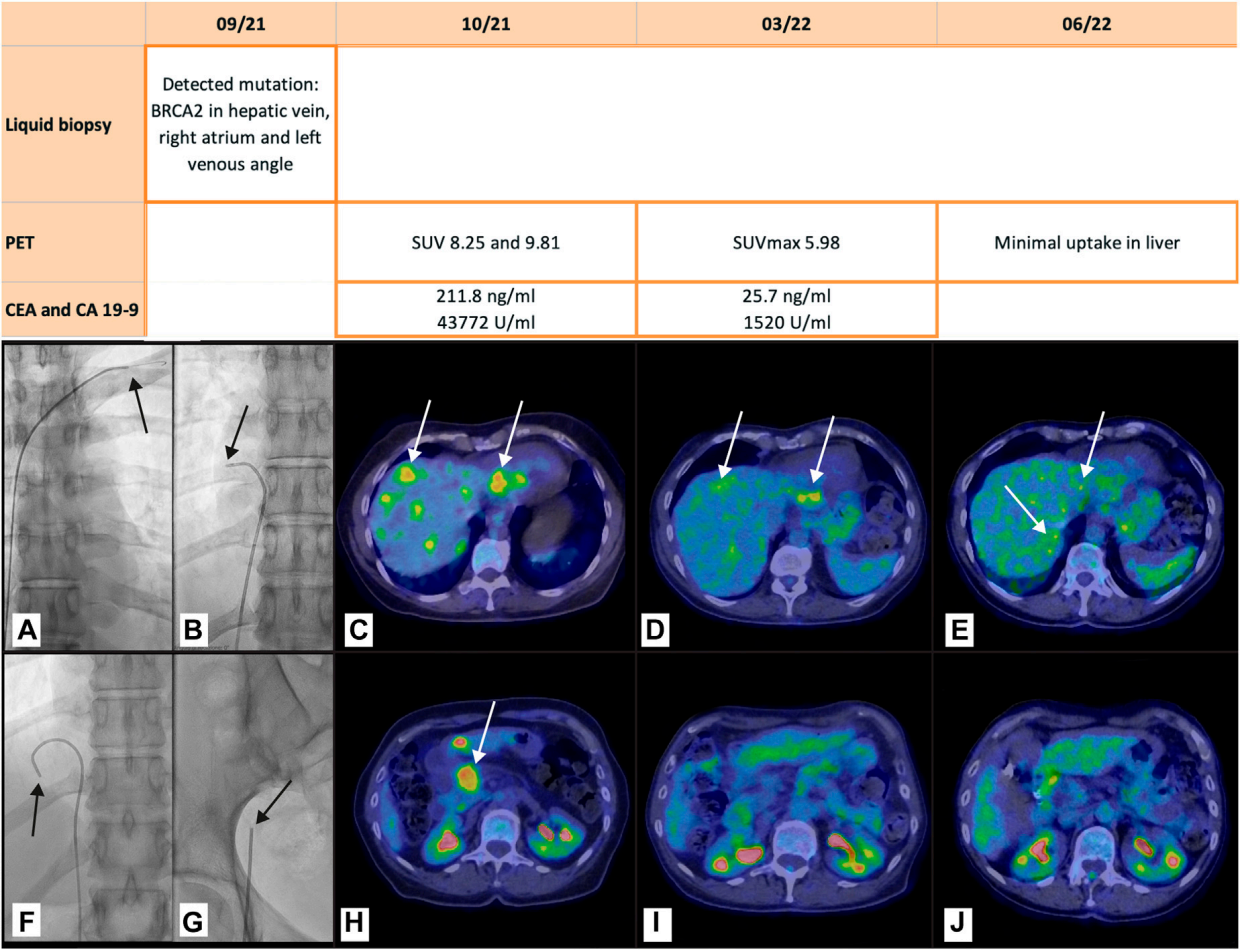
Pancreatic adenocarcinoma with liver metastases. The table shows the tests performed. The figure shows the radioscopic images of the catheter position (arrows) for selective venous sampling in the left venous angle **(A)**, in the right atrium **(B)**, and in the right hepatic vein **(F)**, and for peripheral sampling in the right femoral vein **(G)**. In the PET CT scan at staging **(C, H)**, 5 months later **(D, I)** and 8 months later **(E, L)** the arrows indicate the liver metastases and the primary pancreatic cancer before treatment **(C, H)**, the therapeutic response in the liver, where some lesions (arrows) remain **(D, I)**, and the absence of uptake in the pancreas. At the end of chemotherapy there is still weak uptake in the liver metastases whereas the almost complete response in the pancreas continues **(E, L)**.

A 69-year-old woman in fairly good health with a family history of gastric adenocarcinoma had undergone cholecystectomy at 50 years of age for gallstones. She presented with abdominal pain and an ultrasound diagnosis of cancer of the pancreatic head with liver metastases. Ultrasound endoscopic biopsy confirmed the diagnosis of biliopancreatic adenocarcinoma. CT staging revealed hilar adenopathy and liver metastases. Selective liquid biopsy was suggested in order to obtain a molecular diagnosis. The BRCA2 mutation was found in samples from the right hepatic vein [(c.1813delA, p. (I605fs*9), variant allele frequency (VAF) 7.22% (coverage: 194 reads)], the right atrium [(c.1813delA, p. (I605fs*9), VAF 10% (coverage: 79 reads)], and the left venous angle [(c.1813delA, p. (I605fs*9), VAF 8.33% (coverage: 60 reads)] indicating treatment with platinum drugs and the PARP inhibitor Olaparib. No mutations were found in the peripheral sample. A PET scan subsequently confirmed the CT findings (SUV8.25/9.81). CEA was 211.8 ng/mL, and CA19-9 was 43,772 U/mL. Chemotherapy was begun with six cycles of FOLFIRINOX (Park et al., 2021). Follow-up CT and PET CT showed radiological improvement, which was accompanied by a reduction in abdominal pain and a large decrease in tumor size and in FDG uptake intensity (SUVmax 5.98). In addition, CEA fell to 25.7 ng/mL and CA 19-9 fell to 1,520 U/mL. This objective response continued at follow-up PET CT 3 months later but subsequently the cancer progressed and the patient died of multi-organ failure 13 months after disease onset. Figure 1 shows the course of the investigations, the images relating to the selective sampling method and the PET CT finding of malignant disease.

## Case 2

**FIGURE 2:**
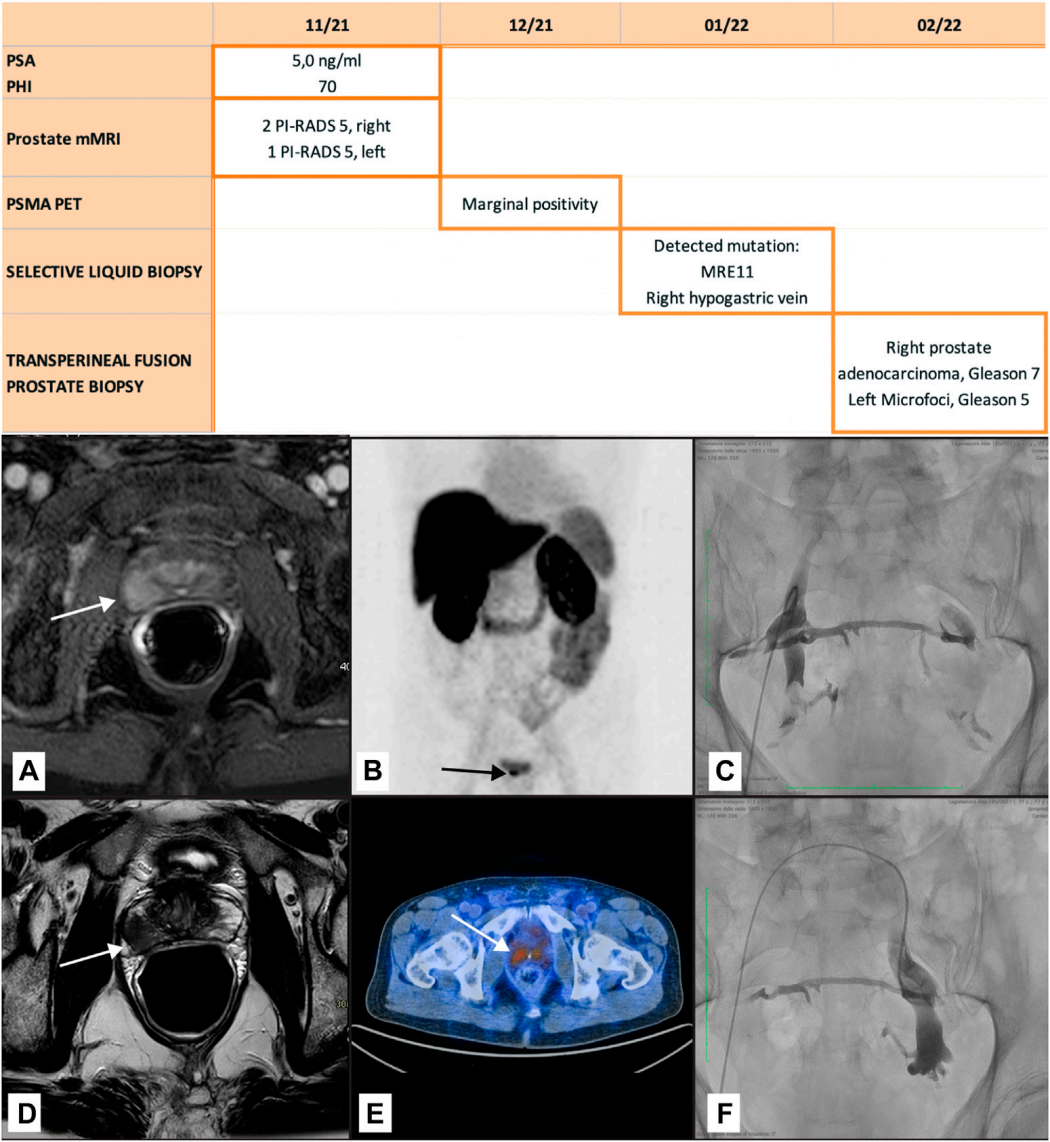
Prostate adenocarcinoma. The table shows the tests perfomed. The figures show the multiparametric MRI detecting the tumor **(B, C)**, the PSMA with marginal positivity **(D)**, and right transfemoral catheterization of the hypogastric veins for ctDNA sampling **(E, F)** with the veins opacified by injection of contrast medium. The two sides are connected by the pelvic venous plexus.

A 65-year-old man with a family history of a brother who underwent surgery for prostate cancer at the age of 63 years underwent urological examination, multiparametric MRI and PSMA (Figure 2) after a finding of raised PSA at 4.63 ng/mL. A subsequent SLB was positive for the MRE11 mutation [(c.685A>C, p. (I229 L), VAF 2.87% (coverage of the variant: 174 reads, exon 8, NM_005591.4)] in the right hypogastric vein but negative in the left hypogastric vein and in the peripheral blood (DNA at the lower limit of detection). Transperineal prostate biopsy confirmed the cancer in the right lobe (Gleason 3 + 4) associated with microfoci in the left lobe. Radical prostatectomy resulted in a diagnosis of bilateral prostate adencocarcinoma (Gleason 3 + 4) infiltrating the apical and lateral extraprostatic tissue. The resection margins were clear, as were the pelvic lymph nodes and the seminal vesicles. Tumor volume was 8% of the parenchyma. None of the above variants were detected on analysis of the parafin-embedded tissue (mean coverage of the analysis: 2362 reads). Figure 2 shows the course of the tests and radiological imaging.

## Case 3

**FIGURE 3:**
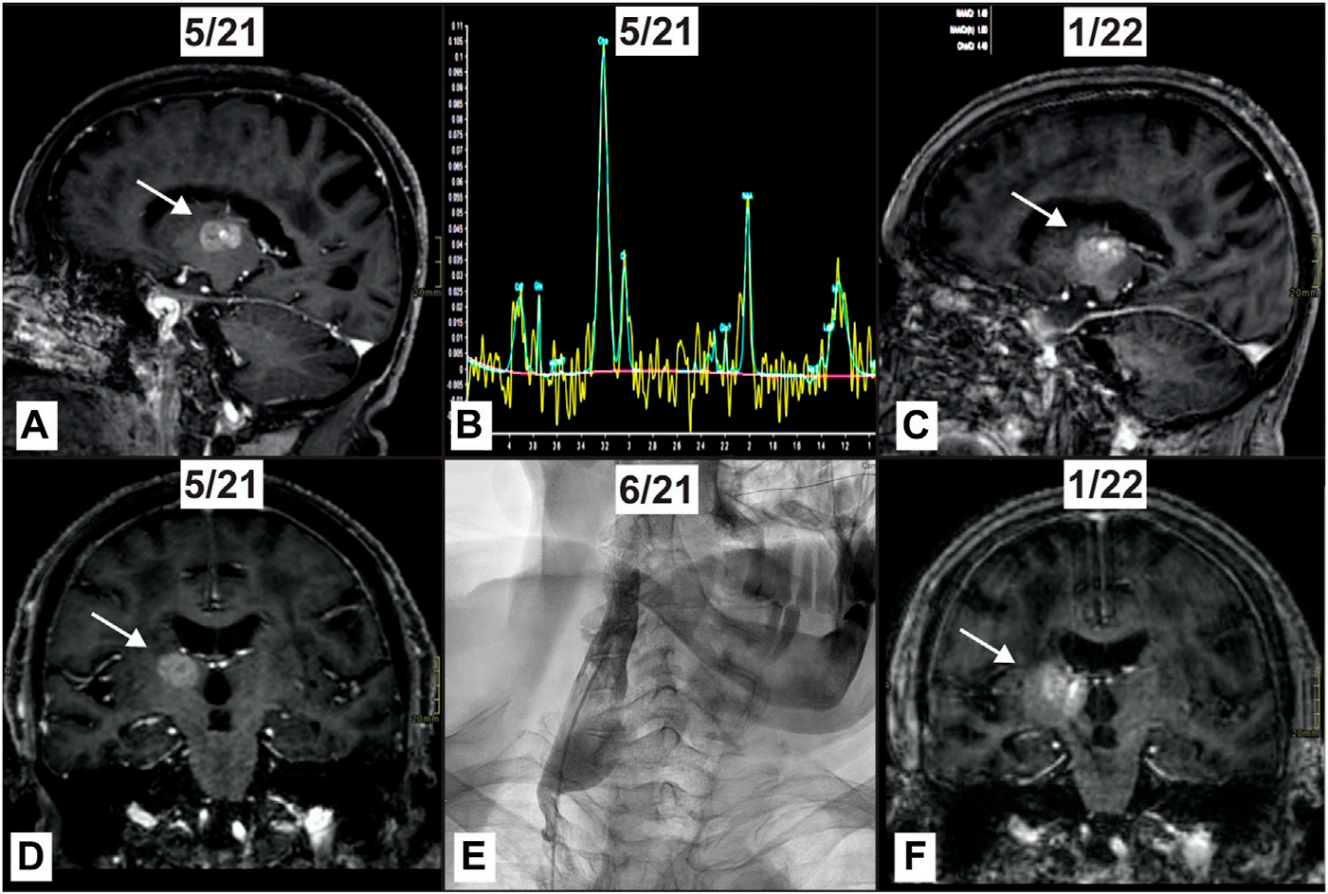
Thalamic tumor in a patient with previously resected pulmonary adenocarcinoma. High field MRI shows an enhancing mass (arrow) within the right thalamus **(A, D)**. MR spectroscopy shows a high peak of Cho with inversion of Cho/NAA and high Cho/Cr ratios with a lipid peak, compatible with the suspicion of a glial tumor **(B)**. Right jugular vein where sampling has been obtained detecting ALK mutation variant **(E)**. High field MRI acquired 8 months later reveals the mass (arrow) has increased in size **(C, F)**.

An 81-year-old woman with dyslipidemia, hypertension, hypothyroidism and paroxysmal atrial fibrillation, who was a current smoker with an unremarkable family history, had received a radiological diagnosis of left lung cancer at age 76 years during cardiology follow-up. The diagnosis was confirmed by PET CT, which showed ipsilateral hilar adenopathy with no evidence of extra-thoracic disease at staging. She underwent left upper lobectomy and hilo-mediastinal lymph node resection resulting in a diagnosis of pT2 pN2 pulmonary adenocarcinoma. Adjuvant chemotherapy was ruled out on account of her age and her cardiovascular disease, but she received adjuvant mediastinal radiotherapy at 50.4 Gy. At age 79 years, a follow-up total body CT revealed a solitary indefinite lesion in the right thalamus which was interpreted as a possible cavernous angioma. Follow-up 6 months later showed a slight increase in size, justifying a contrast-enhanced MRI which failed to clarify the diagnosis. The patient was neurologically asymptomatic. Brain MRI 3 months later showed no change in the lesion. After an oncology consultation, selective liquid biopsy was recommended. A mutation in the ALK gene [c.3824G>A p. (Arg1275Gln) VAF 6.74% (coverage: 89 reads) MN_004304.5], associated with primary brain tumor (Webb et al., 2009), was detected only in the sample from the right jugular vein. To support this diagnostic impression, a molecular diagnosis was carried out on the histology specimen from the previously resected pulmonary adenocarcinoma. This showed an EGFR mutation [c.2303_2304insTGTGGCCAG p.A767_V769dup (VAF 31%)], frequently found in non-small cell lung cancer (Qin et al., 2020), but no evidence of the gene mutation found in the SLB sample (Kalamatianos et al., 2018). Radiotherapy was ruled out because of the SLB result suggesting a glial tumour at a site inaccessible to histological investigation. In addition, the patient was by now exhibiting a deterioration in her general health associated with cognitive decline. A further MRI of the brain with MR spectroscopy supported the diagnosis of glial tumour. The patient subsequently began to experience epileptic fits, which were pharmacologically controlled, and died of heart failure at age 82 years with no further symptoms. Figure 3 shows the imaging of the brain lesion and the selective sampling method involving retrograde catheterization of the right jugular vein.

## Discussion

Liquid biopsy represents a shift in tumor characterization from the microscopic to the molecular. However, the analytical and clinical validity of this rapidly developing diagnostic method remains to be confirmed.

Using a single percutaneous access, SLB can reach numerous body regions, depending upon the site of the primary cancer or metastases. In the absence of radiological evidence of tumor locations, sampling sites are chosen based on our knowledge of tumor spread along blood and lymph vessels. Alongside the advantage of a greater concentration of ctDNA, selective sampling provides topographic mapping of disease distribution. The mutational heterogeneity characteristic of tumor evolution can be tracked more readily and less invasively than with repeated incisional or excisional biopsies. The three cases reported here highlight the potential advantages of SLB over peripheral LB in clinically complex cases, in which SLB can provide important diagnostic information.

Case No. 1 suggests SLB as a molecular diagnosis to provide possible treatment indications. In this case of adenocarcinoma of the pancreatic head with liver metastases diagnosed histologically by ultrasound endoscopy there was too little tissue for a molecular diagnosis. Despite the extensive disease, the BRCA2 mutation was not detected in the peripheral blood sample, in our opinion because of the dilution of the biological maker in the general circulation. The choice of the most appropriate chemotherapy was confirmed by the significant objective response, which was accompanied by an improvement in the patient’s quality of life and ultimately by longer survival. In this specific case, if only the peripheral sample had been relied upon, the chemotherapy regime suggested by the presence of the mutation might not have been adopted, exposing the patient to toxicity without a correspondingly greater efficacy.

The choice of sampling sites took into account the venous drainage from the pancreatic lodge towards the portal circulation and thence into the hepatic venous system. Catheterization of the right hepatic veins is straightforward, but in order not to lose any contribution of the left hepatic veins, which are smaller and more difficult to identify in the upper third of the inferior vena cava, a sample can be collected from the right atrium. Lastly, for abdominal cancers, we suggest collecting a sample from the left venous angle, into which the thoracic duct carries lymph from the subdiaphragmatic compartment. Lymph and the interstitium form a single compartment, a preferential route for cancer spread (Benias et al., 2018).

Case No. 2 shows how prostate cancer, a hormone dependent tumor, has diagnostic and therapeutic peculiarities (Casanova-Salas et al., 2021) which mean that molecular diagnosis could bring a shift in the management of this condition. The MRE11 mutation was found only in blood drawn from the right hypogastric vein, corresponding to the tumor site revealed by MRI, whereas the absence of mutations in the left hypogastric vein was not in agreement with the prostate biopsy result which showed microfoci on the left. In this case too the negative result of the peripheral sample highlighted the appropriateness of SLB, which held the additional advantage of highlighting the resistance to radiotherapy expressed by the mutation (Wang et al., 2021).

In case No. 3 SLB was used for the differential diagnosis of a thalamic lesion detected radiologically during follow-up after curative treatment of lung cancer. This was a unique opportunity for SLB since tissue biopsy of thalamic lesions is difficult or even inadvisable. To the best of our knowledge, this is the first time that onset and development of a glial tumor have been seen in a relatively rare site, not accessible for tissue biopsy. This case justifies a more favorable view of those factors that might be considered cause for criticism of SLB compared with peripheral sampling. It is a short procedure that is minimally invasive for the patient and can be readily accessed in catheterization laboratories and interventional radiology units. The high current cost of SLB due to the number of samples for processing is usually warranted by the indication.

## Conclusion

Analysis of ctDNA with detection of the somatic mutations specific to a tumor is complementary to microscopic diagnosis on tissue but brings with it treatment indications that cannot otherwise be obtained because of the genetic heterogeneity of tumors. Tissue biopsy can be difficult, as in the case of brain tumors, in pediatric oncology, in deep organs, and in at-risk patients. Moreover, it does not have a role in follow-up after presumed radical surgery. Liquid biopsy offers the prospect of revising therapeutic practice based on conventional staging. The possibility of demonstrating or excluding minimal residual disease will mean that patients erroneously deemed to be cured can be treated and those that really are cured can avoid exposure to unnecessary toxicity. In addition, liquid biopsy can detect recurrence after primary treatment before it is clinically or radiologically evident, informing selection of molecular targeted therapy (Coombes et al., 2019).

Critical issues in molecular analysis on blood or other body fluids hinder the current application of liquid biopsy because of the large number of false negatives (Rolfo et al., 2020). The minimal amounts of ctDNA released into the circulation are largely responsible for the diagnostic failures of liquid biopsy. We believe that liquid biopsy with selective venous sampling could prove of value in clinically complex cancer patients to whom it can be offered as an alternative or a complement to traditional tissue biopsy. The utility of introducing analysis of the biological markers released into the circulation as part of the diagnosis and follow-up of the most common cancers remains to be confirmed in large-scale studies (Heidrich et al., 2020). Likewise, the diagnostic advantage of SLB as proposed here requires confirmation in a larger number of cases in order to justify the invasiveness, though minimal, of percutaneous venous catheterization. In the meantime, individual cases such as those reported here and in previous work (Damascelli et al., 2021) may help to evaluate the appropriateness of possible methodological changes aimed at improving the sensitivity and specificity of liquid biopsy.

## Data Availability

The raw data supporting the conclusion of this article will be made available by the authors, without undue reservation.
